# Infarct quantification with cardiovascular magnetic resonance using "standard deviation from remote" is unreliable: validation in multi-centre multi-vendor data

**DOI:** 10.1186/s12968-022-00888-8

**Published:** 2022-11-07

**Authors:** Einar Heiberg, Henrik Engblom, Marcus Carlsson, David Erlinge, Dan Atar, Anthony H. Aletras, Håkan Arheden

**Affiliations:** 1 Department of Clinical Sciences Lund, Clinical Physiology, Skåne University Hospital, Lund University, 222 42 Lund, SE Sweden; 2 Wallenberg Center for Molecular Medicine, Lund University, Lund, Sweden; 3 Laboratory of Clinical Physiology, National Heart, Lung, and Blood Institute, NIH, Bethesda, USA; 4 Department of Cardiology, Skåne University Hospital, Lund University Hospital, Lund University, Lund, Sweden; 5 Department of Cardiology, Oslo University Hospital Ullevål, University of Oslo, Oslo, Norway; 6 Institute of Clinical Medicine, University of Oslo, Oslo, Norway; 7 Laboratory of Computing, Medical Informatics and Biomedical-Imaging Technologies, School of Medicine, Faculty of Health Sciences, Aristotle University of Thessaloniki, Thessaloniki, Greece

## Abstract

**Background:**

The objective of the study was to investigate variability and agreement of the commonly used image processing method “n-SD from remote” and in particular for quantifying myocardial infarction by late gadolinium enhancement (LGE) cardiovascular magnetic resonance (CMR). LGE-CMR in tandem with the analysis method “n-SD from remote” represents the current reference standard for infarct quantification. This analytic method utilizes regions of interest (ROIs) and defines infarct as the tissue with a set number of standard deviations (SD) above the signal intensity of remote nulled myocardium. There is no consensus on what the set number of SD is supposed to be. Little is known about how size and location of ROIs and underlying signal properties in the LGE images affect results. Furthermore, the method is frequently used elsewhere in medical imaging often without careful validation. Therefore, the usage of the “n-SD” method warrants a thorough validation.

**Methods:**

Data from 214 patients from two multi-center cardioprotection trials were included. Infarct size from different remote ROI positions, ROI size, and number of standard deviations (“n-SD”) were compared with reference core lab delineations.

**Results:**

Variability in infarct size caused by varying ROI position, ROI size, and “n-SD” was 47%, 48%, and 40%, respectively. The agreement between the “n-SD from remote” method and the reference infarct size by core lab delineations was low. Optimal “n-SD” threshold computed on a slice-by-slice basis showed high variability, n = 5.3 ± 2.2.

**Conclusion:**

The “n-SD from remote” method is unreliable for infarct quantification due to high variability which depends on different placement and size of remote ROI, number “n-SD”, and image signal properties related to the CMR-scanner and sequence used. Therefore, the “n-SD from remote” method should not be used, instead methods validated against an independent standard are recommended.

**Supplementary Information:**

The online version contains supplementary material available at 10.1186/s12968-022-00888-8.

## Introduction

Late gadolinium enhancement (LGE) cardiovascular magnetic resonance (CMR) is currently considered the reference standard for in vivo assessment of infarct size [1]. Even though LGE is considered the reference standard for infarct quantification there is still no consensus on which quantitative method to use for quantifying infarction in LGE images [2, 3]. In the latest available consensus document from the Society for Cardiovascular Magnetic Resonance (2020) Task Force refrains from making a dedicated statement regarding the optimal method for infarct quantification: “As the research applications are evolving and consensus evidence is being accumulated, the Task Force chooses to refrain from making a dedicated statement at this time regarding the optimal method for quantitative assessment” [2, 3]. The quest for an accurate and precise standardized method is important for studies of efficacy of treatment, and pathophysiology studies. Without such standardized method results from different studies cannot be compared.

There are several methods for LGE infarct quantification including manual planimetry, Full Width Half Maximum (FWHM) [4, 5] Otsu’s method [6, 7], expectation maximization (EWA) [8], Gaussian mixture model classification [9], and level set methods [7]. The most common method in the literature is the use of a fixed number of standard deviations “n-SD from remote”. This fixed number is anything between 2 and 8, which suggests that this methodology has limited reliability. This approach has also been proposed for quantification of several aspects of myocardial injury, such as infarction [10–12], Myocardium at Risk (MaR) [13, 14], and gray zone (region of intermediate signal intensity within the infarcted region which has shown prognostic value for predicting ventricular arrhythmia and need for implanted cardiodefibrillator (ICD) [15].

The aim of this study was to establish the performance of “n-SD from remote” for quantifying infarction by LGE CMR and to analyze sources of error associated with this methodology.

This study is divided into two parts, first an evaluation of the mathematical theory behind the concept of “n-SD from remote” to find the theoretical basis and potential source of variation for infarct quantification. The second part of the study provides clinical data on different sources of errors from two multi-center, multi-vendor cardioprotective trials.

## Theoretical analysis

**Fig. 1:**
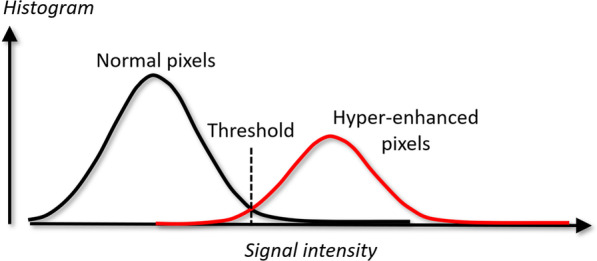
Discrimination of hyper-enhanced pixels to non-enhanced pixels. The graph shows two histograms of non-enhanced pixels (black) and hyper-enhanced pixels (red). The x-axis depicts signal intensity (arbitrary units), and y-axis number of pixels

The image processing task of infarct quantification, within the endocardial and epicardial borders, is to determine for each pixel in the myocardium if it should be considered as non-infarcted or hyper-enhanced (infarcted). The basis for this decision is the brightness of the pixel compared to pixels defined to constitute non-infarcted myocardium. To do this, classification statistical methods are used to compute a threshold to discriminate hyper-enhanced pixels from non-infarcted pixels. This discrimination task is illustrated in Fig. 1.

The pixel intensities within the non-infarcted remote myocardium are assumed to be described by a normal distribution, which is described by the mean *µ* and the standard deviation *σ* (width of the distribution). The normal distribution is often used to describe if a parameter falls outside what is considered a “normal range”.

**Fig. 2:**
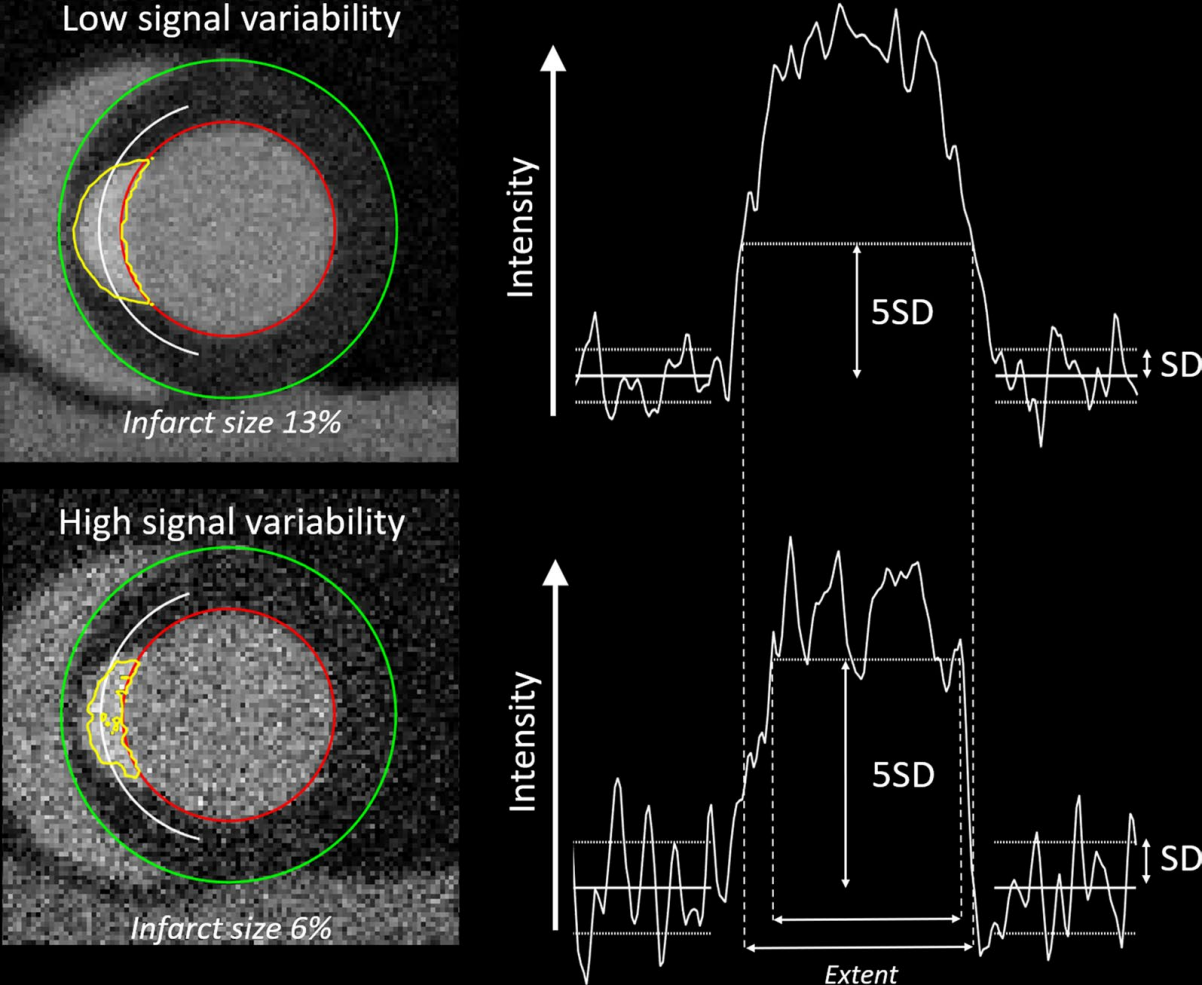
Illustration on how signal variability in remote region-of-interest (ROI) influences infarct quantification in a mathematical phantom. Given a fixed SD from remote (in the example 5 SD) a high signal variability in leads to infarct size underestimation, whereas low signal variability leads to overestimation. The white line indicate where the signal intensity curve is extracted from

With the “n-SD from remote” method the observer manually defines a region of interest (ROI) in the non-infarcted part of the myocardium (remote region). Inside that region, a normal distribution of signal intensities is assumed and the mean (*µ)* as well as standard deviation (*σ)* are estimated. These are then used to compute a *threshold* between non-enhanced pixels and hyper-enhanced pixels as:

$$threshold = \mu + n\sigma$$

where *n* is the number of standard deviations used. Different standard deviations have been proposed, usually in the range between 2–5, for infarct quantification in LGE images [12]. However, standard deviations up to 8 have also been used [16]. Consequences of infarct quantification using “n-SD from remote” in a situation where there is high signal variability or low signal variability are illustrated in a mathematical phantom in Fig. 2. For the same infarct, the measured infarct size is smaller and underestimated in the case of high signal variability, whereas it is larger and overestimated in the case of low signal variability.

This threshold is applied to the entire myocardium and pixels above the threshold are designated as hyper-enhanced. Thus, the method relies on both the estimated mean signal intensity and the standard deviation within remote myocardium. By applying a threshold the resulting segmentation often contains spurious pixels. In some publications such spurious pixels have automatically been removed. However, many studies fail to mention whether this is done or not. In this study both variants are studied.

In addition, in LGE images microvascular obstruction appears dark because the contrast agent cannot reach the core of the infarct. Such areas will not be detected by the “n-SD from remote” algorithm, which therefore will then underestimate the infarct size. In multiple previously proposed methods various image processing methods to automatically detect microvascular obstruction have been proposed [4, 17].

The “n-SD from remote” method, like all infarct quantification methods, relies on the endocardial and epicardial contours. The effect of endocardial and epicardial contours on the infarct size has previously been studied by Klem et al. [18], and was found to be a major source of error. In order to determine the effect of “n-SD from remote” this source of error was not considered in the present study where endocardial and epicardial contours were kept unchanged.

### Placement and size of remote region of interest

On a per-slice analysis, identifying the remote region implies that there is a remote region without infarction in the same slice. This is not always the case, e.g. in the apical left ventricle (LV) in patients with left anterior descending (LAD)-infarctions where the infarction often involves the entire circumference, leaving no possibility to define any remote myocardium. Therefore, this study focused on a per-slice analysis in order to study the intrinsic behavior of the “n-SD method” without assuming consistent intensity profiles across slices. The placement of the remote ROI and the size of the ROI are user dependent. The larger the remote ROI is, the smaller the statistical error in sampling the true underlying signal intensity variation. However, the larger the remote ROI is, the higher the risk of including image artifacts. Furthermore, the remote ROI will have a different mean intensity and intensity variation depending on its position due to inhomogeneities in receiver coil sensitivity.

### Myocardial signal intensity variation

The signal intensity variation in the remote region should not only be interpreted as imaging noise as there is an underlying variation in physiological signal intensity from i.e. myocardial vasculature and interstitial space. Measuring and understanding imaging noise or variability is complex in the setting of parallel imaging [19], an acceleration technique that is used in most implementations of LGE sequences across vendors and scanners. The variability in signal intensity will differ between LGE pulse sequences due to variations in field strength, coil placement, body size, reconstruction techniques, receiver bandwidth, parallel imaging, coil loading, as well as potential out-of-spec coil elements. The signal intensity variability also depends on different characteristics such as noise suppression techniques employed by the scanner’s vendor in acquisition and image reconstruction. Even for the same vendor, signal intensity variation depends on how well remote myocardium has been “nulled” by choosing the correct inversion time. Finally, the “n-SD from remote” method assumes that the pixels in remote myocardium are normally distributed (Gaussian). The noise characteristics for a properly nulled myocardium in a magnitude LGE image (i.e. when the mean value is close to zero) are not Gaussian. In a setting of magnitude images and multiple receiver coils the distribution is non-central chi and in a single coil the noise follows a Rician distribution [20].

### Dependence on number standard deviations from remote used

The impact of “n-SD from remote” on infarct quantification is relatively well studied [12, 16, 21–24]. When the distributions between remote myocardium overlap with the intensities of the infarct the choice of “n-SD” from remote will influence the result significantly. If the hyper-enhanced pixels are well separated from non-infarcted myocardium with little overlap, the actual number “n-SD” is less critical. However, this is often not the case in clinical LGE images.

## Methods and materials

This section contains clinical data on the sources of error observed with the “n-SD from remote method” based on the theoretical analysis in part one*.*

### Patient population and image analysis

Subjects from two multi-center, multi-vendor prospective cardioprotective trials, CHILL-MI [25] and MITOCARE [26], were included. In short, the inclusion criteria for both trials where first time ST-elevation myocardial infarction and the patients were scanned on day 2–6. Infarction in all three coronary vessels were included. The two trials included data from 19 sites from 6 different European countries. One site included only one subject with adequate LGE images and these were excluded in the analysis. The vendor distribution of the CMR scanners was (37% Siemens), (50% Philips), and (13% General Electric). All studies were performed on 1.5 T CMR systems. Details on the imaging protocol are included in Additional file 1: Table S2.

Image analysis in the included clinical trials was performed using the software Segment [27]. For all subjects, infarct quantification was performed by the core lab (Imacor AB, Lund, Sweden) using a validated algorithm [17]. In short, the algorithm automatically finds an infarct threshold and performs processing to remove spurious pixels. It also involves and processing across slices to support completely infarcted slices. To decrease the potential impact of an incorrect infarct threshold the algorithm uses a weighted approach where the infarct size is weighted based on the signal intensity. The algorithm was validated with computer phantoms, in an animal setting and in patients. In the core lab analysis careful manual corrections were performed where needed. All observers were level 3 European Association of Cardiovascular Imaging (EACVI) CMR certified, and all delineations had a second opinion. For challenging cases, the final delineation was adjudicated in consensus.

**Fig. 3:**
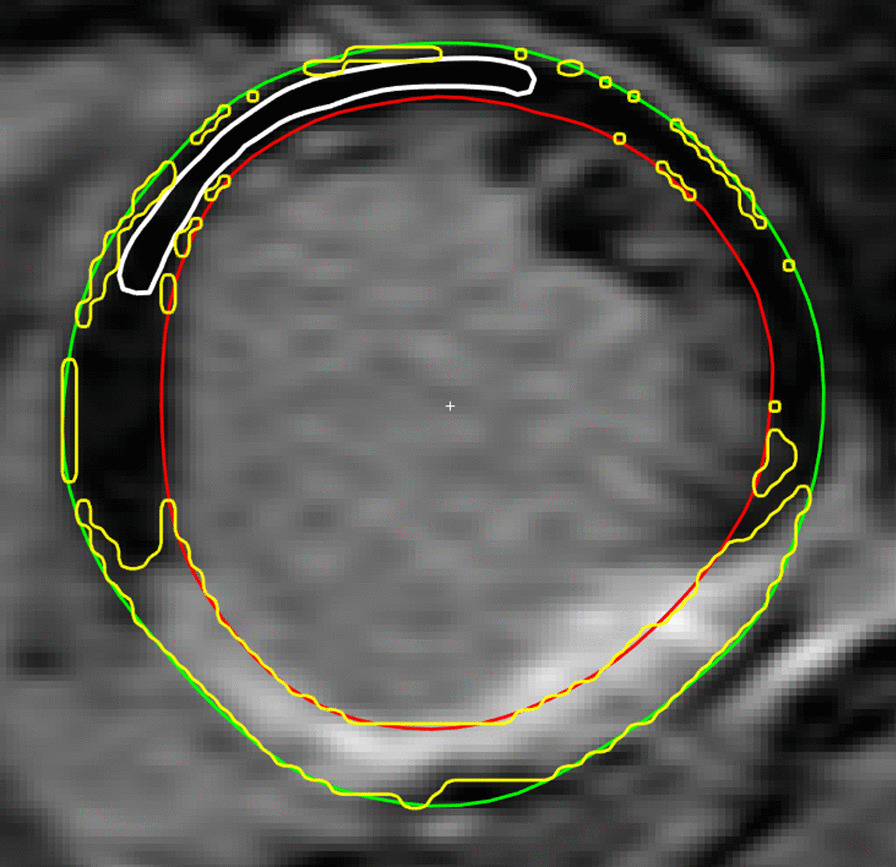
Example of automatic placement of ROI (white) in remote region. Yellow indicates delineated scar region using the “n-SD from remote” method with n = 5. Note that the threshold is applied to the entire myocardium leading to non-physiological infarct regions close the endocardium and epicardium

All slices that contained ≥ 10% of infarction circumference and had at least a 50% contiguous circumference without infarction according to core lab consensus delineation were included for analysis. Different ROIs were placed in the part of the myocardial circumference marked as “remote myocardium” i.e. not containing any infarction. All remote ROIs were automatically drawn by a computer algorithm and excluded the 15% of the endocardial and epicardial side, respectively. Example of automated drawn remote ROI and quantification with “n-SD from remote” is illustrated in Fig. 3. The ROIs excluded most of the endocardial and epicardial pixels. Two versions of the “n-SD from remote” were implemented; the first, named *naïve,* did not remove spurious pixels and the second, named *no-spurious,* did remove all spurious pixels in disconnected regions that consisted of less than 10 pixels.

To study the impact of image quality, one observer (HE) performed quality scoring of all images (1 = Poor image quality, 2 = Adequate image quality, 3 = High image quality). Note there were no studies of unacceptable image quality as they had already been excluded by the core lab from any analysis in the original trials. Images were analyzed all together but also in subsets of different image quality.

Five sources of errors were studied:

- *Impact of remote ROI placement: *A fixed size ROI consisting of 45 degrees of the circumference was placed in different locations over the region marked as infarction-free by the core lab. The position was shifted in steps of at least 10 degrees so that, if possible, up to 7 different ROI positions could be considered for each slice.
- *Impact of remote ROI size: *A set of 7 different sized ROIs were placed in the region limited by the part of the circumference that was marked as remote myocardium and free of infarction by core lab consensus.
- *Impact of the number “n-SD” from remote used: *A fixed size ROI consisting of 45 degrees of the circumference was placed diametrically opposed to the region marked as containing infarction by the consensus delineation. The number “n-SD” from remote used was varied within common ranges in literature as n = 2 to n = 8 in steps of 1 (i.e. for a total of 7 steps).
- *Impact of spurious pixel removal: *To investigate the impact of spurious pixel removal two versions of the “n-SD from remote” algorithm were implemented, one naïve and one with spurious pixel removal.
- *Impact of image quality: *The “n-SD from remote” algorithm with spurious pixel removal was evaluated on all studies together but also in subsets of studies with high, adequate, and poor image quality. For each run the mean error (accuracy) and variability (precision) was evaluated separately when changing ROI placement, ROI size, and number “n-SD”.

Variability was expressed in percentage units and computed as the coefficient of variation, i.e. standard deviation of the error divided by the reference infarct size. Note that the slices were chosen so that reference infarct covered more than 10% of the circumference, which ensured that the normalization did not introduce a large variability. Accuracy was computed as absolute difference in median of the infarct size to consensus core lab infarct size.

In addition, the following descriptive statistics across vendors, sites, and subjects were computed:

- *Comparison of optimal number “n-SD” across vendors, sites, and subjects*
- For each slice the number “n-SD” from remote was adjusted to get as close as possible to the reference delineation of infarction. A fix size remote ROI consisting of 45 degrees of the circumference was used.
- *Comparison of signal variability (σ) between vendors, sites, and subjects*
- For the first set of experiments, where remote ROI placement was varied, the signal intensity variability was measured and compared between vendors, sites, and subjects. Signal intensity variability was measured in arbitrary units where each image was normalized between 0–1 and the signal intensity variability was computed as the standard deviation of signal intensity in the remote ROI.

### Statistics

All statistics were performed in Matlab (R2019a, Mathworks, Natick, Massachusetts, USA. Results are presented as median and interquartile ranges.

## Results

In total 214 research subjects from two cardioprotective trials were included. Of these subjects, 196 had LGE with interpretable image quality. In a number of studies, LGE imaging was not performed due to technical reasons or because the research subject declined contrast. Eighteen (9%) studies were scored as poor quality, 111 (57%) studies as adequate, and 67 (34%) as high quality. The reference infarct size was 17 ± 10% of LV mass and the microvascular obstruction was 1.9 ± 3.6% of LV mass. A total of 1268 slices met the inclusion criteria of at least 10% of the circumference with some infarction and at least 50% of contiguous circumference with no infarction. The mean infarct percentage per slice was 18%. The accuracy and precision for the different versions of the algorithm and different sets of the data are presented in Table 1.

**Fig. 4:**
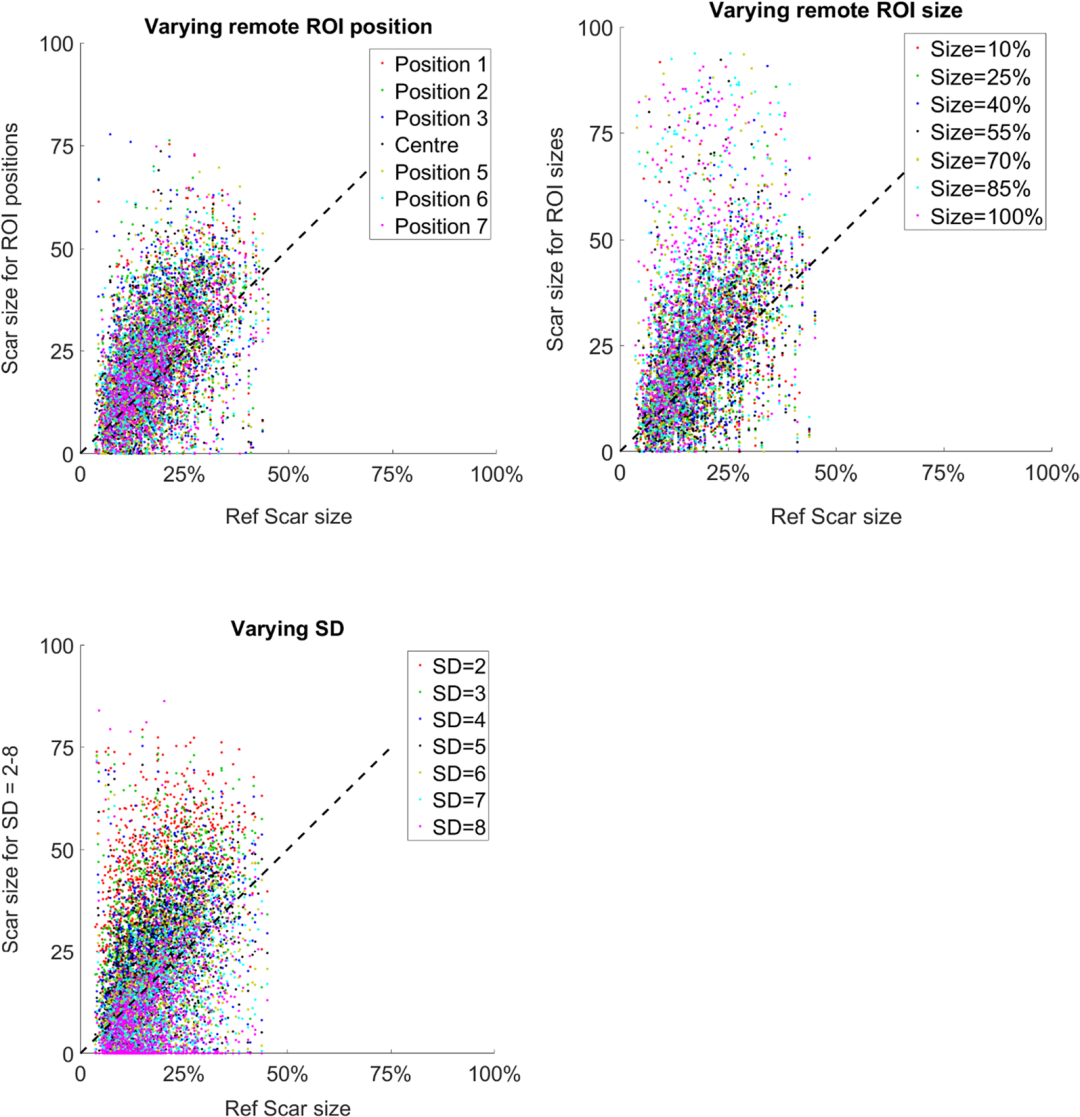
Accuracy of “n-SD from remote” method illustrated as scatter plot with reference infarct size on X-axis, and infarct size in percent of the slice for different ROI position, ROI size, and number of SD. The dashed line indicates line of identity. Note the large variability compared to reference scar size. The reason that reference is smaller than 50% is that only slices with no more than 50% of infarct circumference were included

The agreement compared to manual consensus delineation is plotted for variations of ROI position, ROI size, and number of SD in Fig. 4. Note the large variability and lack of agreement. The figure is also available as an animation (Additional file 1: Fig. S1). Accuracy was evaluated as error and computed per slice as difference between measured infarct size minus reference infarct size. Variability was computed as the standard deviation of the error divided by the reference infarct size.

**Fig. 5:**
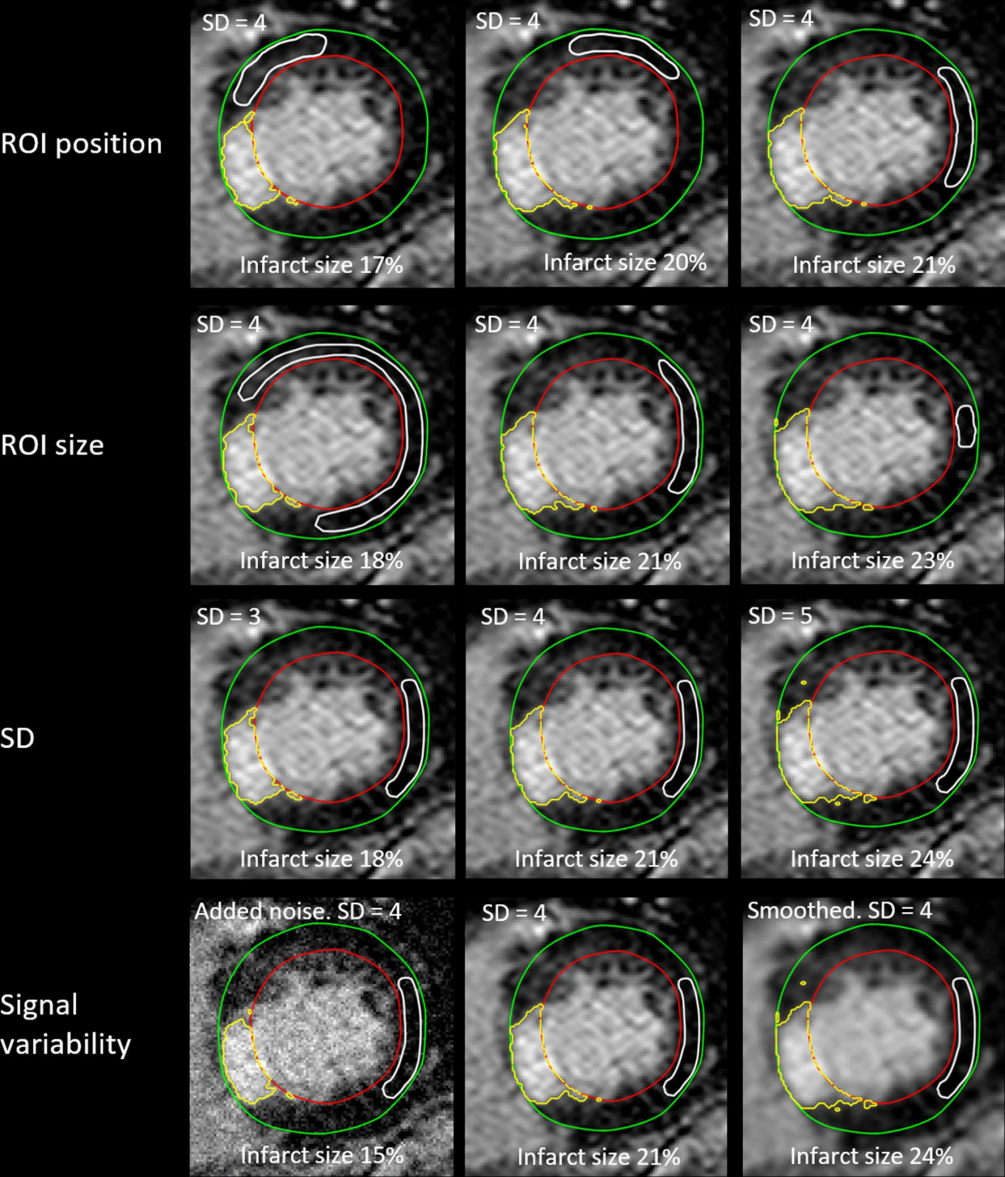
Illustration of the effect of changing ROI-position, ROI size, number of standard deviations, and signal variability in a clinical case. The estimated infarct size ranged from 15–24%, which corresponds to a range of 71–114% of the reference infarct size which was 21%

Figure 5 shows an example on a clinical case when varying ROI position, ROI size, number of SD from remote, and signal variability. The estimated infarct size ranged from 15–24%, which corresponds to a range of 71%-114% of the reference infarct size which was 18%.

### Impact of remote ROI placement

The position of the remote ROI could be varied in 1268 slices and in total 8632 region of interests were analyzed (slices with infarct < 10% were excluded from analysis). Varying ROI placement when the ROI size was kept fixed to 45 degrees and the number n of SD was fixed to 3.5 generated an overestimation of 8 ± 6 percentage units. The variability, defined as coefficient of variation, was 47% ± 46%.

### Impact of remote ROI size

The size of the remote ROI could be varied in 1244 slices resulting in 7990 ROIs. Varying ROI size while keeping ROI placement fixed in the middle of the remote region and the number n of SD fixed to 3.5 generated an overestimation of 5 ± 9 percentage units. The variability was 48% ± 72%.

### Impact of the number “n-SD” from remote used

The number “n-SD” from remote used was varied from 2 to 8 in 1265 slices resulting in a total 8855 infarct segmentations. Varying the number n of SD from remote used between 2–8 (while keeping ROI placement fixed in the middle of the remote region and ROI size fixed to 45 degrees) generated an overestimation of 4 ± 13 percentage units. The variability was 61% ± 42%.

### Comparison of optimal number “n-SD from remote”

In total 1265 slices the number of “n-SD” from remote was varied on a slice per slice basis to get as good agreement as possible to reference infarct. The resulting “n-SD from remote” was 5.3 ± 2.2. Data are also presented per site in Additional file 1: Table S2.

### Comparison of signal variability (σ) between slices, subjects, and vendors

**Fig. 6:**
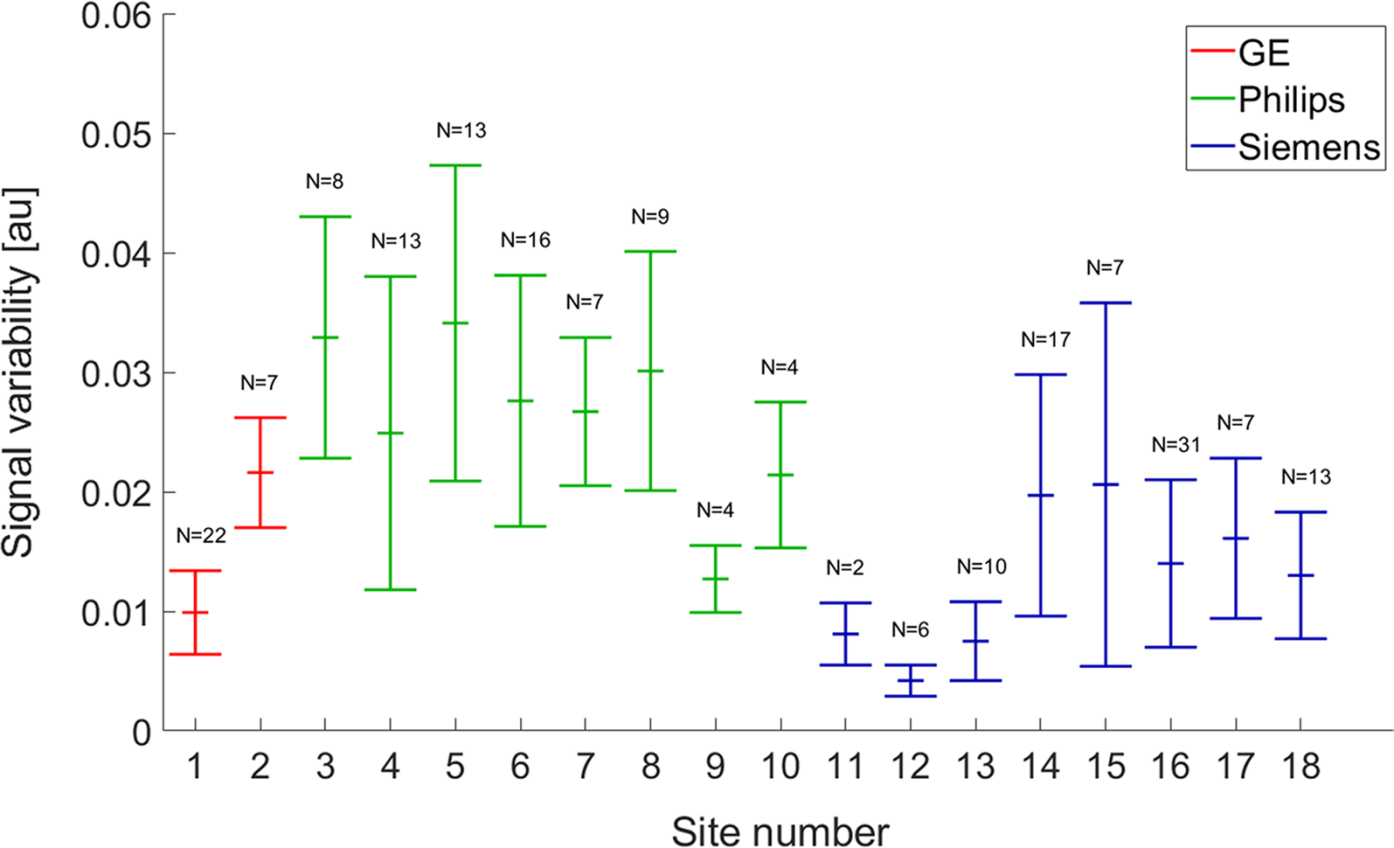
Signal variability in remote region of interests for sites and vendors. Bars indicate mean and SD across subjects for the different sites. Number on top of each bar represents the number of patients included in the study with sufficient infarct for usage in this study. Site 4 & 8 is the same physical site from the two different cardioprotective trials

Signal variability in different regions was 0.016 ± 0.008, 0.026 ± 0.004, 0.013 ± 0.006 for General Electric, Philips, and Siemens scanners, respectively (Fig. 6).

## Discussion

Infarct size quantification using LGE CMR is heavily affected by position and size of the ROI and the number of SD chosen when performing image analysis with the “n-SD from remote” method. Considering the high variability, other post-processing methods less dependent on the signal intensity characteristics of the remote ROI should be used for infarct quantification to decrease the sample size needed in trials using LGE-CMR.

The agreement between the “n-SD from remote” method and reference infarct size by core lab delineations in consensus was poor (Fig. 4). The importance of removing spurious pixels was small with a reduction of one percentage unit in infarct size. Removing spurious pixels did not affect variability. The image quality affected the measured infarct size. In images with worse image quality on average the measured infarct size was smaller than in images with better quality. This is consistent with Theory as described in Fig. 2.

Interestingly, the variability caused by changing ROI position, ROI size, and number n- SD from remote used was about the same. The “n-SD from remote” number of standard deviations to use has been extensively studied and discussed in existing literature, whereas little attention has been paid on how to place the remote ROI and what ROI size to use, which this study clearly shows have a profound impact. The mean variability for the sources of variability from ROI position, ROI size, and “n-SD” were 47%, 48%, and 40%, respectively. As an example, with a 50% variability, a true infarct size of 15% of LVmass would result in estimated infarct sizes from 7.5 to 22.5%.

Different “n-SD from remote” numbers from remote to be used on LGE images have typically been proposed in the literature ranging from 2–5 and, in one case, up to 8 [16]. Thus, there is no consensus on the number “n-SD from remote” to be used. This can be explained by the different vendors’ implementations of the LGE pulse sequences and settings used by different sites performing the examination.

In the literature several studies have reported agreement with manual delineations using 4 or 5 SD [12]. This could not be confirmed in the present study, nor in a previous study by Engblom et al. [8]. Of note, previous studies were single center studies and our work was performed on data from multicenter studies using CMR scanners from three vendors.

Difference in signal variation between vendors should not be interpreted as one vendor having better coils, amplifiers, but rather attributed to different noise removal algorithms employed during image reconstruction and perhaps due to differences in pulse sequence parameters, and proficiency at the different sites on nulling the remote myocardium. The large signal intensity variability in the remote region between vendor, sites and individual patients shows that not a single best “n-SD” from remote can be defined. Even for the same vendor, signal intensity variation depends on how well remote myocardium has been “nulled” by choosing the correct inversion time, which also may vary with renal clearance of the contrast agent during the examination.

The data analyzed in the present study include images from all three major vendors and were acquired at 19 sites. Two of the sites corresponded to the same physical site, but from two different cardioprotective trials. The signal variability between sites can explain the range of “n-SDs” from remote suggested in the literature since the vast majority of studies have been single-center studies.

In principle, the size of the remote ROI and the number “n-SD” from remote used could be standardized but that would not solve the problem with the method since the signal variability between vendors, sites, and subjects remains. The lower quartile of signal variability was 20% and the upper quartile 55%, which corresponds a change from 2 SD to 5.5 SD. Furthermore, the “n-SD from remote” was not able to handle microvascular obstruction.

The sensitivity to signal variability can partly be mitigated by weighting the voxels intensity with signal intensity [8, 17]. This compensates for partial volume effects as well as limiting the effect of an incorrectly selected threshold.

In agreement with findings in the present study, it has previously been shown that the “n-SD from remote” method for infarct quantification is associated with significant variability [8, 17]. In addition, it has been shown that “n-SD from remote” (with n = 2 to 4) performs worse than non-SD methods regarding infarct quantification [8]. On the contrary, there are no prior studies of the “n-SD from remote” method in a multi-center, multi-vendor setting that show that the method works.

### Can “n-SD from remote” be used in any instance for infarct quantification?

Variability in signal intensity in the remote region between imaging sites and vendors cannot systematically be accounted for. Therefore, the number “n-SD” from remote used cannot be the same for all subjects, even from the same site. A per patient-specific “n-SD” is feasible, however, it will closely correspond to the case where an observer manually outlines the infarct region. Thus, single-center studies where the ROIs are manually drawn by an experienced user and “n-SD” adjusted, so that the resulting delineation would correspond with the visual impression of the infarction, may be accurate. Multi-center studies or studies where the ROIs have been automatically drawn and with a fixed “n-SD” should be interpreted with great caution.

### Validated methods for infarct quantification

Any method for infarct quantification should be validated for both accuracy (bias) and variability against an independent objective method. To our knowledge, there are only a few methods that have been validated against an independent standard (FWHM [4] [5], weighted method for partial volume [17] and EWA [8]. To our knowledge, the only data published to date in a multi-center, multi-vendor setting are those from Engblom et al. [8], which show that both EWA and FWHM seem to be feasible in such settings.

It is important to stress that no automated method is “perfect” and there should always be a manual observer that can correct cases were the automated methods is incorrect.

### Limitations

Our study has several limitations. To compute accuracy and variability, a reference standard is necessary. For this we used consensus delineation by a core lab using a validated method [17] with manual corrections where necessary. Manual segmentation as ground truth can always be debated but is, albeit time consuming, the best available reference standard in patient cohorts. One source of variation that was not evaluated in this study is the effect of endocardial and epicardial contours. This was in detail evaluated by Klem et al. [18] and was found to be a major source of variability regardless of method used (manual, or automated). The “n-SD from remote” method was evaluated without manual corrections to evaluate its underlying performance. However, this may not always be how the method would be applied in practice.

### Generalization

The specific application described in this study is infarct quantification by LGE CMR. The very same arguments presented in this work, however, apply to any other usages of the “n-SD from remote” methodology e.g. for quantifying MaR from T2-STIR images [14], MaR from T1 and T2 images [28], intramyocardial hemorrhage [29], or grey zone [30]. The principles are also valid for all forms of medical image processing where the “n-SD” from remote method is used. The only instance where “n-SD” potentially could be used would be if the noise distribution is Gaussian or quasi-Gaussian and the signal-to-noise ratio is well defined and importantly similar between subjects and sites. These assumptions are not fulfilled for any known medical imaging modality or reconstruction method.

## Conclusion

Infarct quantification in LGE CMR images using the “n-SD from remote” method is not reliable as the variability is approximatively 50% due to variability in placement of remote ROI, size of remote ROI, number “n-SD” from remote, and underlying signal properties of the images depending on site and sequence used. Other image analysis methods with less variability should be used in trials using infarct quantification.

**Table 1:** Results overview

Method	Mean error % units (accuracy)			Variability (CoV) %precision)		
	ROI pos	ROI size	SD	ROI pos	ROI size	SD
Naïve, all data	5 ± 9	7 ± 13	4 ± 13	47 ± 46	48 ± 72	61 ± 42
Spurious removal, all data	3 ± 9	6 ± 13	3 ± 12	43 ± 45	45 ± 72	57 ± 40
Spurious removal, high quality	5 ± 8	9 ± 12	5 ± 11	41 ± 38	46 ± 66	54 ± 37
Spurious removal, good quality	3 ± 9	5 ± 13	3 ± 13	44 ± 50	43 ± 77	57 ± 41
Spurious removal, poor quality	− 3 ± 9	− 1 ± 12	− 2 ± 12	44 ± 38	51 ± 61	62 ± 50

Mean error and variability for the different versions of the algorithm and different sets of the data. Naïve refers to not removing spurious pixels, no-spurious refers to removing spurious pixels. High quality, good quality and poor quality refers to that the analysis was only performed on the data subsets with the corresponding image quality rating. A positive mean error (accuracy) represent overestimation compared to reference infarct size and negative mean error an underestimation. Mean error is expressed in scar percentage units. Variability (precision) is expressed as coefficient of variability, i.e. standard deviation of scar sizes divided by reference infarct size

## Supplementary Information

**Additional file 1: Table S1.** Sequence details for the different sites. **Table S2. **Optimal “n-SD” per site. **Figure S1. **Animated gifs showing correlation between reference infarct size and measured infarct size. A) Impact of ROI position, B) Impact of ROI size, C) Impact of “n-SD from remote”. Note the large impact on bias when changing “n-SD from remote”.

## Data Availability

The software Segment which was used for core lab analysis and scripted analysis is freely available for research purposes at http:/www.medviso.com/segment. The software includes the infarct quantification algorithm presented by Engblom et al. [8]. The extracted image statistics in the current study are available from the corresponding author on reasonable request. Images are not available due privacy concerns and because this was not explicitly included in the ethical permits.

## Abbreviations

EACVI: European Association of Cardiovascular Imaging
CMR: Cardiovascular magnetic resonance
EWA: Expectation maximization weighted algorithm
FWHM: Full width half maximum
ICD: Implanted cardiodefibrillator
LGE: Late gadolinium enhancement
LV: Left ventricle/left ventricular
MaR: Myocardium at risk n-SD: number of standard deviations
n-SD: number of standard deviations
ROI: Region-of-interest
SD: Standard deviation(s)
