# Supplementary material for: Infarct quantification with cardiovascular magnetic resonance using "standard deviation from remote" is unreliable: validation in multi-centre multi-vendor data
Source: J Cardiovasc Magn Reson. 2022 Nov 7;24:53. doi: 10.1186/s12968-022-00888-8 (PMC9639305; doi:10.1186/s12968-022-00888-8)
Supplement: Supplementary file 1 — Additional file 1: Table S1. Sequence details for the different sites. Table S2. Optimal “n-SD” per site. Figure S1. Animated gifs showing correlation between reference infarct size and measured infarct size. A) Impact of ROI position, B) Impact of ROI size, C) Impact of “n-SD from remote”. Note the large impact on bias when changing “n-SD from remote”. [file 12968_2022_888_MOESM1_ESM.docx]

***Table S1****. Sequence details for the different sites.*

| **Site** | **Vendor** | **Type** | **Resolution  [mm]** | **TR  [ms]** | **TE  [ms]** | **FA [^o^]** |
| --- | --- | --- | --- | --- | --- | --- |
| **1** | *General Electric* | *2D* | *1.4* | *4.4* | *1.2* | *15* |
| **2** | *General Electric* | *2D* | *0.8* | *5.3* | *1.5* | *20* |
| **3** | *Philips* | *3D* | *0.8* | *4.0* | *1.3* | *15* |
| **4** | *Philips* | *3D* | *1.4* | *4.2* | *1.3* | *15* |
| **5** | *Philips* | *Mix* | *0.8* | *6.1* | *3.0* | *25* |
| **6** | *Philips* | *3D* | *1.4* | *4.2* | *1.3* | *15* |
| **7** | *Philips* | *Mix* | *0.6* | *6.1* | *3.0* | *25* |
| **8** | *Philips* | *3D* | *1.4* | *4.2* | *1.6* | *15* |
| **9** | *Philips* | *3D* | *1.3* | *5.8* | *2.8* | *25* |
| **10** | *Philips* | *2D* | *0.6* | *4.1* | *1.3* | *15* |
| **11** | *Siemens* | *2D* | *1.4* | *700* | *3.3* | *25* |
| **12** | *Siemens* | *2D* | *1.5* | *700* | *3.3* | *25* |
| **13** | *Siemens* | *3D* | *1.5* | *700* | *1.4* | *10* |
| **14** | *Siemens* | *2D* | *1.3* | *700* | *1.2* | *45* |
| **15** | *Siemens* | *** | *1.9* | *700* | *3.0* | *25* |
| **16** | *Siemens* | *2D* | *1.5* | *770* | *1.2* | *50* |
| **17** | *Siemens* | *2D* | *1.6* | *800* | *1.2* | *45* |
| **18** | *Siemens* | *2D* | *0.8* | *860* | *1.5* | *20* |

***Table S1****. Sequence details for the different sites. Type is what type of sequence, i.e either 2D or 3D sequence. Two sites were not consistent and are reported as mix. For one site we could not determine what type was used based on image information. Resolution refers to reconstructed resolution and is median value of all studies for that site. Repetition time (TR) are given as the median value for studies for that site. Note that Siemens reports TR differently compared to General Electric and Philips. Echo time (TE) are median for studies for that site. Flip angle (FA) is given in degrees.*

**Table S2.** *Optimal “n-SD” per site*

| **Site** | **Vendor** | **Optimal “n-SD”  mean*±SD, (25% and 75%-quartile)*** |
| --- | --- | --- |
| **1** | *General Electric* | *5.1±2.0 (3.6, 6.7)* |
| **2** | *General Electric* | *3.3±1.8 (2.1, 4.1)* |
| **3** | *Philips* | *5.0±1.8 (3.7, 6.2)* |
| **4** | *Philips* | *5.3±2.2 (3.7, 7.5)* |
| **5** | *Philips* | *5.5±2.3 (4.0, 7.8)* |
| **6** | *Philips* | *4.8±2.0 (3.3, 6.4)* |
| **7** | *Philips* | *5.8±1.9 (4.3, 7.4)* |
| **8** | *Philips* | *5.4±2.0 (4.0, 7.5)* |
| **9** | *Philips* | *5.5±1.8 (3.8, 7.3)* |
| **10** | *Philips* | *5.2±1.9 (4.1, 6.6)* |
| **11** | *Siemens* | *4.1±1.6 (3.7, 5.2)* |
| **12** | *Siemens* | *6.3±1.9 (4.7, 7.8)* |
| **13** | *Siemens* | *6.5±1.6 (5.7, 7.8)* |
| **14** | *Siemens* | *5.3±2.0 (3.6, 7.0)* |
| **15** | *Siemens* | *5.2±2.0 (3.7, 6.8)* |
| **16** | *Siemens* | *6.0±2.0 (4.4, 7.9)* |
| **17** | *Siemens* | *5.2±2.0 (3.4, 6.8)* |
| **18** | *Siemens* | *5.3±2.0 (3.9, 7.5)* |

***Table S2****. Optimal “n-SD” per site with mean±SD and (25% and 75%-quartile). Note the large variability in optimal “n-SD” within each site as expressed by the interquartile range. The mean interquartile range was 3 SD-units.*


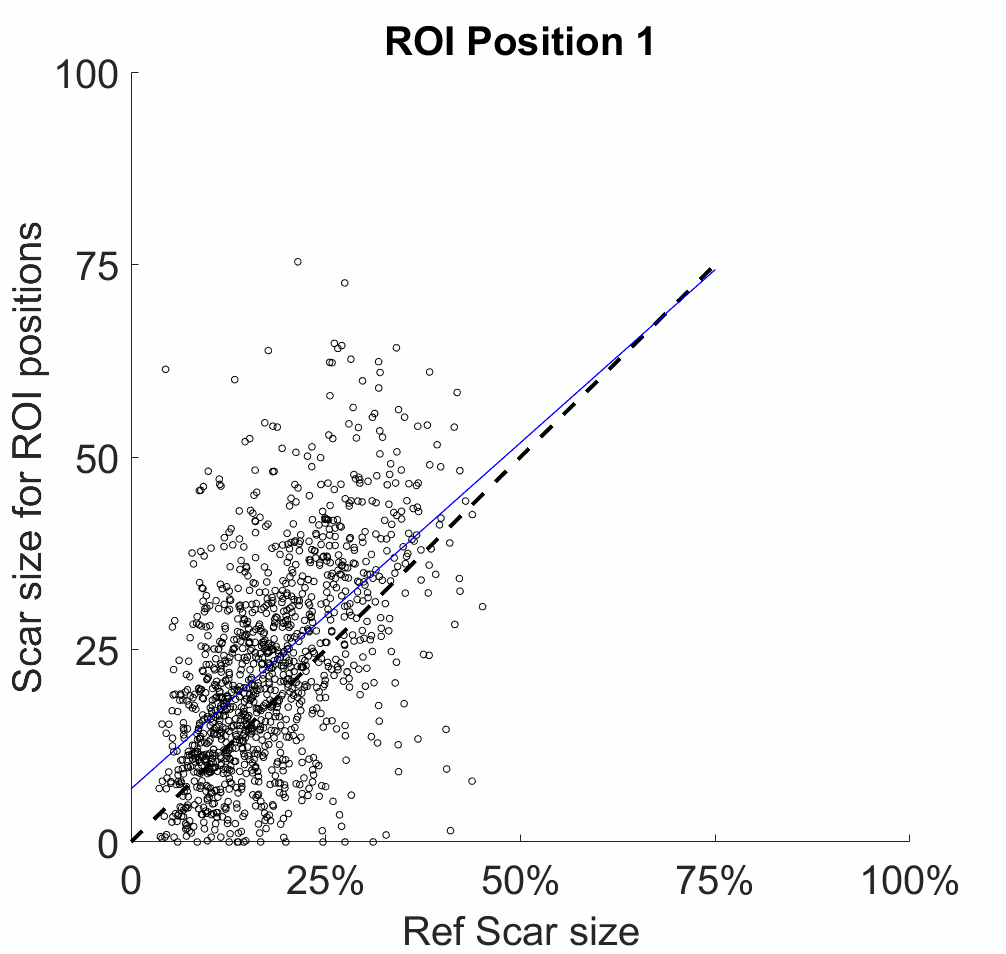

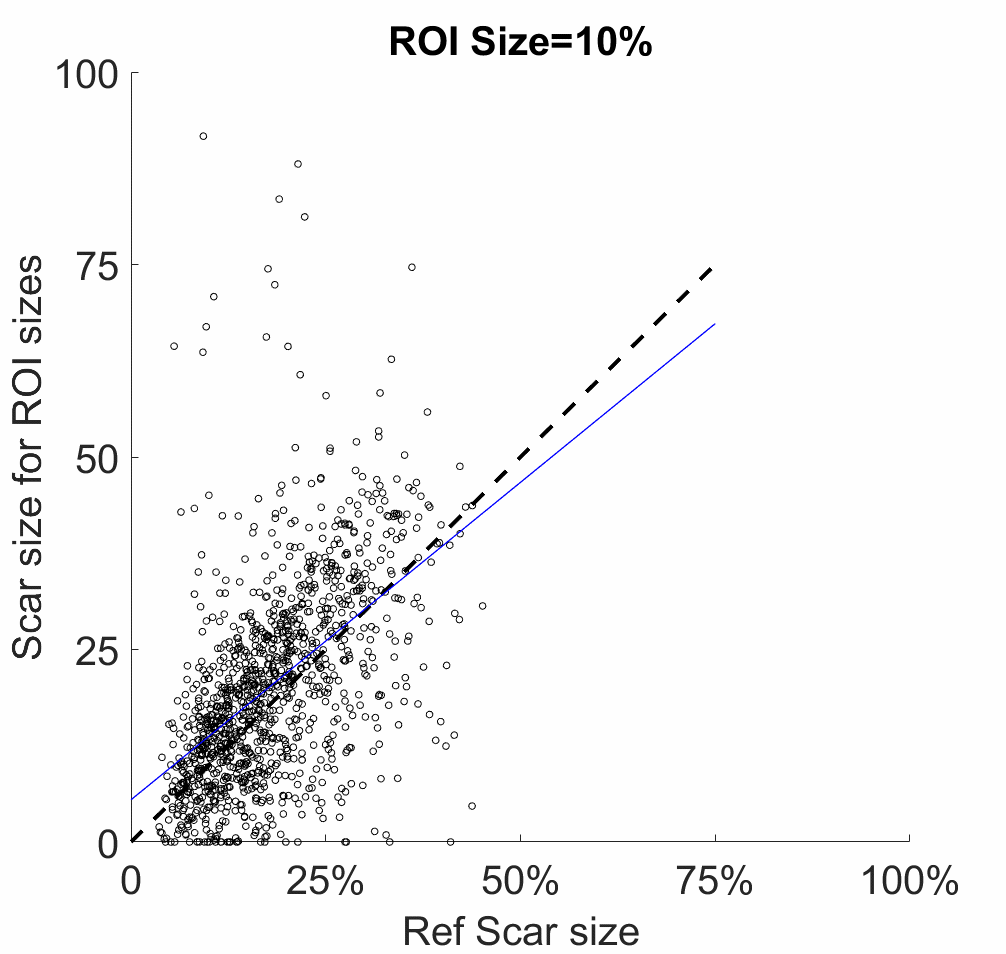

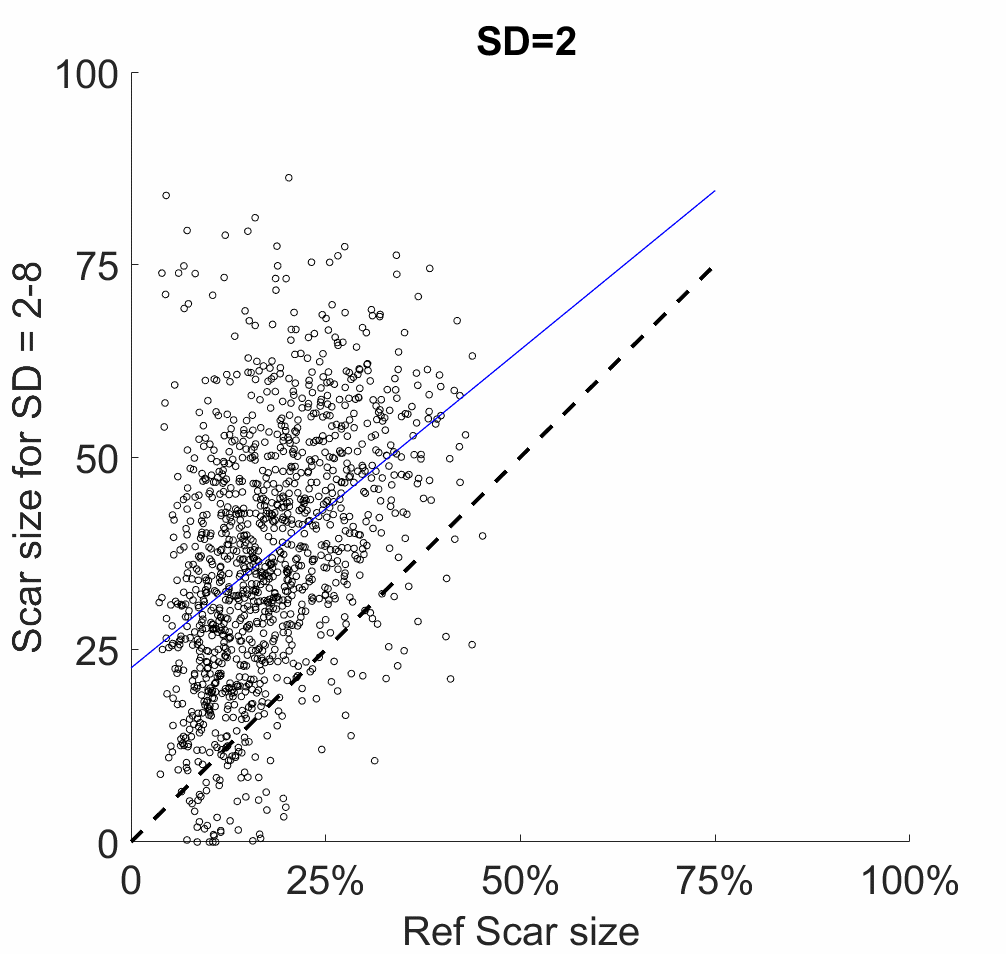


***Figure S1.*** *Animated gifs showing correlation between reference infarct size and measured infarct size. A) Impact of ROI position, B) Impact of ROI size, C) Impact of “n-SD from remote”. Note the large impact on bias when changing “n-SD from remote”.*
